# Determining the destination: a co-designed chronic advanced cancer rehabilitation conceptual framework for engagement of individuals with lived experience in rehabilitation research

**DOI:** 10.1186/s40900-024-00566-2

**Published:** 2024-03-25

**Authors:** Naomi Dolgoy, Stephanie Bernard, Fleur Huang, Amy Driga, Debra Hall-Lavoie, Adam Brown, Edith Pituskin, Alysa Fairchild, Margaret L. McNeely

**Affiliations:** 1 Department of Occupational Therapy, University of Alberta, Edmonton, AB Canada; 2 École des sciences de la réadaptation, Faculté de Médecine, Université Laval, Quebec City, Canada; 3 Department of Radiation Oncology, Cross Cancer Institute, Edmonton, AB Canada; 4 Alberta Health Services, Edmonton, AB Canada; 5 Individuals With Lived Experience, Edmonton, AB Canada; 6 Faculty of Nursing, Department of Oncology, Cross Cancer Institute and University of Alberta, Edmonton, AB Canada; 7 Department of Physical Therapy, Department of Oncology, Cross Cancer Institute and University of Alberta, Edmonton, AB Canada; 8 Cancer Rehabilitation Clinic, University of Alberta, Edmonton, AB Canada; 9 Faculty of Medicine and Dentistry, Department of Oncology, University of Alberta, Edmonton, AB Canada

**Keywords:** Patient-oriented, Patient-engagement, Rehabilitation, Physiotherapy, Occupational therapy, Speech therapy, Oncology, Advanced cancer, Chronic cancer, Metastatic cancer

## Abstract

**Background:**

Individuals living with chronic advanced cancer (CAC) often face distinct physical, functional, and cognitive issues. Their rehabilitation needs are not yet routinely met, warranting further CAC-specific rehabilitation-based research. Given the complexity of functional and symptom presentations, engagement of individuals living with CAC as partners in the research process is encouraged to better understand the lived perspective. Formal engagement requires both structured approaches and iterative processes. The aim was to co-design a conceptual framework to develop and integrate engagement strategies into rehabilitation research focused on CAC populations.

**Methods:**

A multidisciplinary team of authors, including two individuals with lived experience, conducted an implementation-focused descriptive study to inform future research design, including: interviews and follow-up, review of current models and approaches, and development of a co-designed conceptual framework for engaging individuals with lived experience into CAC-specific rehabilitation research.

**Results:**

Emergent themes include shared understanding, transparent appreciation, iterative processes and unique partnership needs. A definition, guiding principles and tools for engagement were identified. In consultation with individuals with lived experience, and application of the emergent themes in context, a conceptual framework to guide the engagement process was developed.

**Conclusion:**

A novel conceptual framework for engaging individuals with lived experience with CAC as partners in rehabilitation research is proposed to facilitate implementation-focused team-based approaches for this population.

## Plain Language Summary

Living with chronic advanced cancer (CAC) affects all parts of a person’s life. Rehabilitation, such as physiotherapy, can be necessary. Healthcare data shows that rehabilitation needs of people with CAC are not yet being regularly met and that more research in this area is needed. Because CAC is complex and impacts each person differently, having people with CAC included as partners on the research team will likely help researchers better understand and explain rehabilitation needs of people with CAC.

Our group of authors include different healthcare professionals, researchers, and two individuals with lived experience. Together, we carried out an implementation study and designed a framework to guide other researchers in including individuals living with CAC on research teams.

We found that important themes for individuals with lived experience were: shared understanding, transparent appreciation, iterative processes (such as back and forth communication) and unique partnership needs.

We titled the conceptual framework a “Co-designed Chronic ADVanced CANCer Rehabilitation” or “Co-ADVANCE” for short.

## Background

Half of North Americans will be diagnosed with cancer in their lifetime [1]. Cancer survivors are commonly considered as individuals who have completed curative-intent therapy, with ongoing care focusing on surveillance [2]. Historically, systemic therapy for many incurable cancers rarely extended life beyond one year [3]. Improved understanding of molecular drivers of malignancy has resulted in exponential medical developments targeting actionable mutations, revolutionizing treatment of many advanced cancers [4]. As a result, an entirely new survivor population has emerged—people living with incurable cancer who are receiving ongoing, recurrent, or episodic treatments [5–7].

**Table:** 

Individuals with lived experience (IWLE) from our groups share that “With early stage cancer you are focused on the goal; getting to the end of treatment, being cured, and moving on with your life. The focus of the patient with chronic cancer centers around trying to stay alive and healthy for as long as you can, while managing all of the side effects that come along with being in treatment for the rest of your life.”	

Individuals live for years with chronic advanced cancer (CAC), requiring regular outpatient visits and self-management around anti-cancer maintenance treatment [7]. Side effects of treatment commonly magnify over time, producing a range of non-uniform and unpredictable toxicities [8]; treatment decisions must balance impacts on symptom burden and function [9].

**Table:** 

IWLE from our groups share that “With improved cancer treatments and care, many of us are living longer. At present, the medical system seems unaware of our long-term survivor experiences. This is likely due to the fact that the health and quality of life issues we are experiencing at five, ten and even twenty years is new territory.”	

Current rehabilitative programming in oncology typically addresses acute impairments or palliative diagnoses [5, 7]. Supportive and rehabilitation-related needs for those living with long-term side effects of CAC are not easily generalizable from existing paradigms [7]. Thus there is a need for evidence on effects of rehabilitation interventions to optimize function and reduce symptom burden in this population.

**Table:** 

IWLE from our groups share that “Even with the most thorough orientation and education, we often wonder ‘is it normal to experience this symptom?’. There is a definite need for broadening the scope of monitoring to better track the complexity of recovery, symptoms, and long-term effects. This may perhaps open the door to earlier identification of recurrence/progression or needed multidisciplinary support for long-term treatment effects.”	

Patient engagement (PE) strategies within the research process provide opportunity for individuals with lived experience (IWLE) to have their concerns and unmet needs addressed through advancements in clinical interventions informed by the research. In a research context PE refers to active participation of IWLE in research processes, including consultive and collaborative roles as informed, involved and empowered members of research teams. PE research approaches may be critical in advancing rehabilitation delivery by involving IWLE with CAC in designing and implementing research interventions [10, 11]. This engagement in research is expected to result in better fit between healthcare provision and those who receive it, ultimately creating more effective and efficient patient outcomes. Despite increases in the breadth of literature on methods for engagement over the past decade, a validated theoretical and practical framework for best methods and impacts of PE in the context of rehabilitation settings is still lacking [12]. A recent systematic review of frameworks for supporting involvement of IWLE and members of the public at large in research included 65 frameworks with different sources, intended purposes, and strengths and limitations [13]. The authors suggest use of a menu of evidence-based resources, utilizable by research teams to co-design their own frameworks, that consider power-sharing, priority setting, patient involvement methods, reporting guidelines, and measures to support partnerships.

With the overarching intent of guiding the integration of PE strategies for future cancer rehabilitation research, the objective of this paper was to co-design a conceptual framework to develop and integrate PE strategies into rehabilitation research focused on CAC populations.

## Methods

### Design

Our multidisciplinary team (including IWLE, researchers, physiotherapists, occupational therapists, nurses, oncologists) completed the following steps: 1) semi-structured interviews, 2) narrative review of PE principles in clinical research, including definitions, published models and frameworks; 3) co-design of a conceptual framework to operationalize patient-oriented oncology rehabilitation research involving IWLE with CAC. Table 1 depicts our team details.

**Table 1:** Team member composition and role

Discipline	Size (N)	Expertise or Experience	Role
Occupational Therapy	2	Research and clinical experience in functional rehabilitation	Authors
Physical Therapy	2	Research and clinical experience in physical functional rehabilitation	Authors
Oncology Nursing	1	Research and clinical experience in oncology nursing	Author
Radiation Oncology	1	Research and clinical experience in medical care	Author
Medical Oncology	1	Research and clinical experience in medical care	Author
Individuals with lived experience of cancer	Group 1: 2 Group 2: 4	Lived experience across: advanced head and neck cancer (*n* = 2), prostate cancer (*n* = 1), and breast cancer (*n* = 1); age groups: < 50 years (*n* = 1); 50–59 years (*n* = 1); 60–69 years (*n* = 1); 70 + years (*n* = 1)	Group 1: formal co-authors; Group 2: consultants for review and feedback

### Step 1) Patient and clinical experiences

Using semi-structured interviews, IWLE with CAC were consulted to identify key concerning issues from their lived experiences with rehabilitative services. Group discussion of clinical experiences identified limitations in how best to support individuals living with CAC for ongoing physical functioning. The perspective of IWLE as members of research teams to inform research was suggested as an essential first step, both in developing this paper, and across research. Results from this first step are apparent through the quotes in this paper and the conceptual framework. These partnerships consisted of two groups. Group one included two individuals who contributed as team members from conception to dissemination; these two individuals (DHL, AB) are living with CAC, having undergone and completed anti-cancer treatments. Group two consisted of four individuals living with metastatic disease and currently undergoing anti-cancer treatments, who acted as consultants, offering iterative independent feedback across the stages of framework development and design.

### Step 2) Narrative review

Narrative review of the literature on definitions, models and frameworks of PE strategies in health research was performed, noting commonalities and gaps within the context of CAC. This review helped identify theoretical foundations of the proposed conceptual framework.

### Step 3) Co-design of a conceptual framework for engagement of IWLE in oncology rehabilitation research

From findings of the previous two steps, an initial proposal for a conceptual framework was developed. All authors shared in conceptualization and development. The agreed upon initial draft was reviewed with group two. Engagement at this step ensured that perspectives of individuals with advanced disease, both current and past, are included in the framework design. Independent feedback was used to adapt and modify the framework, with ongoing iterative feedback provided through our authorship team to reach consensus.

## Results

### Step 1) Patient and clinical experiences

Both groups of IWLE shared their experiences with engagement, in terms of participation in research, and importance of being included in the process and establishment of engagement.

**Table:** 

IWLE from our groups share that “Engagement would mean hat someone who has actually walked the path would help contribute to better care, perhaps contribute to the overall care pathway by providing healthcare experts with the “walked in their shoes” perspective. Survivors can be valuable sources of information to meet this evolving need.”	

Emergent themes are listed with guiding quotations from all groups of IWLE below:

- *Shared understanding*: the importance of being actively engaged in a two-way process with researchers, particularly when clinical research protocols are being developed, was identified. With longer life expectancies and ongoing cancer treatment, IWLE with CAC need to be actively engaged throughout the research process, rather than passively consulted only at the start and/or conclusion. Both groups shared that token engagement only to make the research appear to have patient involvement should be avoided. Examples of solid relationships, as suggested by both groups include: understanding their health challenges, establishing tailored agreements for involvement in research or intervention development; facilitating research partnerships focusing on improvements for future patients.

“Recognize the experience of advanced cancer patients is very unique.”

- *Transparent appreciation*: focusing on recognition in approaches to partnerships and contributions to ensure IWLE are comfortable in their roles and celebrated as part of teams. Both groups suggest this may include forms of compensation, authorship, and/or transparent acknowledgement of time and effort that individuals expend in research engagement.

“We want to clearly understand upfront, how and what the research is attempting to measure, what specifically is required and expected from us.”

- *Iterative processes*: Both groups identified the need to feel like embedded research team members, rather than peripheral members. They suggest relationship and trust building approaches to PE require iterative processes, with ongoing dialogue to ensure that processes continue to be beneficial for all team members. Unique to CAC, both groups of IWLE identified the need for consideration of the disease prognosis and their ongoing health in establishing roles and requirements.

“Frank discussion upfront on suitability to participate: are you well enough? It is important to ask regularly how we are doing; we may not tell you otherwise.”

- *Unique partnership needs of the CAC population*: Engagement research presents opportunities for a spectrum of roles for IWLE, including principal investigators piloting the research. However, in the CAC population, the roles presented as unique and potentially counter to the overarching principles of engagement research; both groups reported preference for reduced roles on the research team, sharing that while they felt they could provide “lived experience”, health-limitations impacted directive engagement. This emergent theme was extremely valuable in explaining the distinct nature of CAC, and the need for engagement to match health-related status.

“We may not want to ‘fly the plane’ but could serve as navigators in determining the destination.”

### Step 2) Narrative review—patient engagement theoretical framework

Through narrative review and development of a theoretical framework, our multidisciplinary team identified 1) a definition for PE, 2) guiding principles for PE, and 3) tools for planning, conducting and evaluating the impacts of PE.

1. *Definition for PE:* Varying nomenclatures, purposes, theories, and definitions across major health research funding bodies blur the literature on PE in research [11, 14–18]. In 2020, Harrington et al. conducted a systematic review of definitions of PE in research [19]. The authors propose adoption of a consensus definition of PE in research:

“The active, meaningful, and collaborative interaction between patients and researchers across all stages of the research process, where research decision making is guided by patients’ contributions as partners, recognizing their specific experiences, values, and expertise.” (Harrington, 2020; pg.682)

**Fig. 1:**
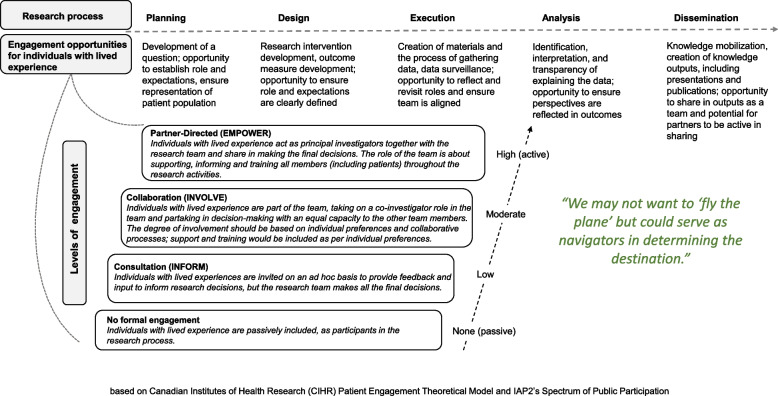
Engagement across the research continuum

2. *Guiding Principles for PE:* Three guiding principles were determined. A first guiding principle underlying the proposed framework is that PE strategies should be applicable to all, or as many, research activities as possible. IWLE can assume varying roles in the research team, i.e., they can collaborate from setting research priorities to disseminating data. At the research planning and design stages, their input ensures that their needs are incorporated into the research agenda, and that research priorities focus on valid patient concerns [20]. Their engagement will influence the final design of outcomes and interventions, facilitate recruitment and data collection, and enrich data analysis. At the final stages of research, they can co-develop implementation and dissemination strategies. A second guiding principle for the framework is that options for varying degrees of commitment should be planned and made available. By displaying the different levels of engagement to choose from, there is less chance that individuals will self-exclude from working on the project because of poor fit. Frequent communication is recommended to validate and reassess that levels of engagement continue to be desirable and achievable. Figure 1 highlights the levels of engagement across the research continuum, showcasing lower versus higher levels of PE and corresponding roles [21]. A third guiding principle for this framework refers to best research practices for Equity, Diversity and Inclusivity [22]. Proactive consideration of the accessibility of the research setting, resources, and equipment available, in addition to a review of recruitment materials’ language and geographic distribution, are examples of recommended strategies to avoid systematic exclusion of potential engagement partnerships. Figure 1 includes a guiding quotation from group two.

3. *Tools:* For rehabilitation research in CAC, two frameworks, one evaluation tool and one checklist were identified: the Patient Engagement in Research (PEIR) Conceptual Framework, the FIRST (facilitate, identify, respect, support, train) Model, the Public and Patient Engagement Evaluation Tool (PPEET) and the Guidance for Reporting Involvement of Patients and the Public (GRIPP) Checklist. PEIR extends beyond guiding principles of engagement, providing guidance and practical details on aspects of engagement in clinical research to ensure meaning for partnerships [23]. FIRST was developed to guide stakeholder involvement in health research at levels of patient and healthcare professionals, given its focus on practical components that facilitate equal collaboration and shared decision-making between IWLE and researchers [24]. PPEET evaluation tool was utilized to assess engagement of individuals, projects and organizations [12]. The GRIPP and GRIPP2 checklists were utilized for planning engagement to ensure comprehensive process and reporting [13]. Both short and long versions of GRIPP2 aim to further the evidence on patient engagement in context. Table 2 reports on our utilization of PE via the GRRIP2 checklist.

**Table 2:** GRIPP2 checklist

Section and Topic	Item	Page No
Aim	The aim of involvement of IWLE in this paper is to develop a co-designed conceptual framework to integrate engagement strategies into rehabilitation research among those living with chronic advanced cancers. We aimed to involve two groups of partners through all the research steps using a Collaboration level of engagement.	P. 6
Methods	• Group one of IWLE were recruited to collaborate on this project. Using semi-structured interviews, they were initially consulted to identify key issues of concern relating to their experiences that would inform the design of the conceptual framework. • The same initial two individuals then collaborated on the conceptualization and development of the initial framework in partnership with the researchers, providing feedback to adapt some words and items, and took part in the consensus process. • Group two included four individuals who participated in the iterative process of reviewing and ensuring that the perspectives of individuals with chronic advanced cancers were included in the design of the framework, providing feedback and suggestions on the words used and items that were missing. • Both groups were involved in the final consensus process for the final version of the framework. • Group one were involved in the writing and editing of the manuscript and are co-authors.	P.5–6
Results (Outcomes from patient involvement in the study)	• From the semi-structured interview, unique and emergent themes were identified and were presented in the results section (shared understanding, transparent appreciation, iterative processes). Those themes were integrated in the design of the conceptual framework. • Resulting from the collaborative and iterative processes with both groups, a co-designed conceptual framework was developed. • Highlighting the experiences of individuals living with and affected by chronic advanced cancer was enabled by the quotations provided by the both groups.	P.7–9 Fig. 2 P.4–5, 7
Discussion and Conclusion	Involvement of both groups influenced greatly the outcomes of this work, especially since they were involved at all stages of research. Every step was planned, undertaken and reviewed in collaboration. We believe this leads to the development of a framework that has a high potential of applicability in upcoming research in this field since it represents the perspective of this population.	P.13–17
Reflections/ Critical Perspective	We identified that there were several strengths related to the involvement of IWLE in the writing of this paper, namely through the use of a stringent scientific approach (semi-structured interviews, narrative review) in gathering information; through shared knowledge from a multidisciplinary team of authors, and through iterative process of feedback and adaptation to reach consensus.	P. 16–17

### Step 3) Conceptual framework for engagement of IWLE with CAC

**Fig. 2:**
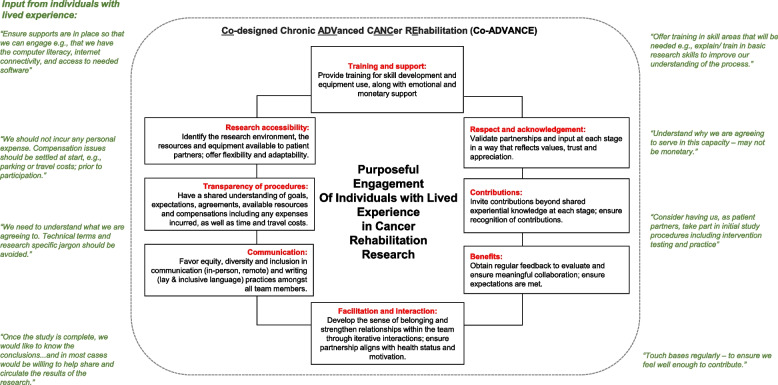
Proposed Co-designed Chronic ADVanced CANCer REhabilitation (“Co-ADVANCE”) conceptual framework, with guiding input from individuals with lived experience

Figure 2 showcases our conceptual framework, titled “Co-designed Chronic ADVanced CANCer REhabilitation” or “Co-ADVANCE”, designed using a collaborative approach, wherein current models were used, and authors were asked to provide iterative feedback throughout the process. Figure 2 includes guiding quotations from both groups.

The Co-ADVANCE framework combines current frameworks, a rehabilitation lens, theoretical constructs, and input from experiential, clinical, and research expertise. Co-ADVANCE focuses on unique needs of the CAC population, such that flexibility and adaptability are crucial, given the ever-changing nature of disease and symptom-burden within this population. The framework underwent four rounds of review within our team, ensuring it clearly reflects an appropriate direction for PE across rehabilitation research in cancer care.

As Fig. 2 depicts, the following key elements (in no particular order) are considered essential to teams developing PE parameters within rehabilitation research: training and support, respect and acknowledgement, contributions, benefits, facilitation and interaction, communication, transparency of procedures, and research accessibility.

## Discussion

With limited PE in CAC rehabilitation research to draw from, reflection on engagement experiences and evidence from other areas of health research offer a foundation to explore and address this gap. Engaging IWLE with CAC throughout the rehabilitative care continuum—starting with early identification of needs and priorities, followed by development and implementation of research approaches addressing those needs, to ultimately clinical delivery of related services at the end of the pathway—has the potential to reinforce the voice of IWLE along all aspects of the rehabilitative care continuum [25]. The Co-ADVANCE framework aims to guide upcoming oncology rehabilitation in engaging IWLE with CAC as research partners.

### Preparation and readiness

Despite limited literature specific to engagement within the CAC population, there is empiric evidence from other aspects of patient and cancer care, as early as the research planning phase [26, 27]. Introspection must be carried out by members of the research team before starting collaborations with patient partners; this reflection allows researchers to identify potential obstacles that could limit or hinder partnerships. Potential barriers can be found in several areas, such as choice of wording, resource allocation, and task management. By addressing those obstacles a priori, research teams minimize IWLE’s need for accommodations or restrictions on their ability to contribute fully and meaningfully. For instance, as consequence of ongoing cancer treatments, CAC patients often deal with various physical impairments, peripheral neuropathies, fatigue, memory loss, communication difficulties, etc. Planning carefully how to best welcome and support IWLE with CAC requires favoring open and transparent communication about health and wellbeing, targeting equity, diversity and inclusion, and empowering IWLE throughout their engagement. The benefits will be mutual for partners and researchers, including diverse inclusive teams with potential for richer research innovation [28].

### Lived experience and public involvement

Early engagement of IWLE allows for different approaches to conducting research through experiential expertise [29]. Building equitable relationships between researchers and IWLE creates multifaceted teams, ensuring that research and dissemination approaches are meaningful to the population being explored and meet that populations’ needs. It also ensures that IWLE have opportunity to contribute according to their expectations and abilities. The different possible levels of engagement need to be offered, supported, and respected for meaningful contribution and good fit.

### Execution

With research activities having potential to benefit from involvement of IWLE, PE strategies can be carefully planned throughout research stages. Specifically, health issues may force unexpected changes in availabilities or team membership, and treatment needs may be less chronological than in acute or palliative cancer populations [30]. Team composition must therefore be viewed dynamically and checked routinely [30]. Biases and power imbalances, real or perceived, must be proactively addressed to avoid unintended marginalization, tokenism, and to ensure that both new and existing patient partners are effectively engaged [10, 26, 31]. Frequent and open communication ensures that patient partners develop a sense of team belonging and remain fully informed and engaged; however, communication must be balanced with selection of convenient approaches. Meaningful engagement is built on shared interactions, celebrated uniqueness, and mutual trust [15, 26, 32–36]. As such, preference for communication (oral, virtual, telephone or written), must be discussed to foster security and comfort, promoting true openness and valuable exchanges amongst members. For example, IWLE on this paper preferred a combination of written responses and 1:1 communication, and so meetings were scheduled to best fit their preferences and needs. Namely, supporting IWLE’s time and expertise goes beyond offering accommodations and availability of support staff; it should involve appropriate financial consideration of compensating time, any expenses incurred, and contribution to the research [37].

### Dissemination: Implementation through engagement

In CAC populations, implementation must coordinate and communicate mechanisms, drawing on cross-cutting and intersectionality [38–40]. Patient partners become part of the research collective [10]. As engagement often aligns with implementation principles, patient partners support the transition of research to clinical contexts [27]. Notably, IWLE have cancer and clinical experiences to share—in doing so, they give voice and perspective to applicability of research into clinical practice, and validation of research relevance. They can contribute beyond their experiential knowledge of disease and treatment; dissemination strategies can be reinforced by their other skills, including: writing and communication abilities, knowledge of the community, and relationships and networks. Pairing implementation research with engagement models or frameworks offers great potential in ensuring partnerships are maintained as vital to research, and engagement continues throughout the process, aligning healthcare delivery goals, rehabilitation, and research. Emergent literature supports the adoption of models of practice and approaches that consider IWLE with CAC as integral team members [41, 42]. Our project is an example of using the PE approach to develop tools for implementation into research practice.

### Assessing PE strategies

Effective PE strategies should be measurable processes leading to positive research outcomes. Essentially, when supporting engagement of IWLE with CAC, formal measurement and reporting of engagement processes, including recognizing partnership value and contributions, are vital to maintaining quality engagement throughout research [26, 43]. Because of the sparsity of PE methods in cancer rehabilitation research with IWLE with CAC, it is essential that future research contribute to developing the evidence base in this field. A measurement tool to assess engagement experience also allows research teams interested in integrating PE practices to consistently evaluate processes, showing awareness of shortcomings, and readjusting throughout the stages of research [12].

## Strengths and limitations

Strengths of this research include: involvement of IWLE with CAC in process and writing; use of a stringent scientific approach (semi-structured interviews, narrative review) in gathering information; shared knowledge from a multidisciplinary team of authors; iterative process of feedback and adaptation to reach consensus. A number of limitations are acknowledged including the scarcity of research and published literature on engagement of IWLE with CAC relative to rehabilitation interventions, and variability in both engagement and CAC terminology.

## Clinical relevance

Further clinical research application of the Co-ADVANCE framework is necessary; we intend to pilot test Co-ADVANCE in upcoming studies, and share the findings and usability of our proposed framework amongst researchers focusing on CAC and rehabilitation interventions.

## Conclusion

Currently, limited partnerships involving IWLE with CAC present in rehabilitation research. Including IWLEwith CAC as research partners promotes planned research relevant to and prioritized by those living with CAC. The proposed Co-ADVANCE conceptual framework for engaging IWLE with CAC in rehabilitation research is intended to guide future research, increasing opportunities for engaging and empowering IWLE to navigate their roles through research and dissemination.

## Data Availability

All relevant models and materials are presented within the manuscript; for transparency, patient-participant quotations have been included throughout the manuscript and within the figures.

## Abbreviations

CAC: Chronic advanced cancer
IWLE: Individuals with lived experience
PE: Patient Engagement
